# Assessment of Functional Capacity and Fatigue Severity in Young Women With Polycystic Ovary Syndrome: A Cross-Sectional Study

**DOI:** 10.7759/cureus.104468

**Published:** 2026-03-01

**Authors:** Satani Kalpesh, Janavi Sanghani, Palani G Kumar

**Affiliations:** 1 Cardiorespiratory Physiotherapy, College of Physiotherapy, Sumandeep Vidyapeeth Deemed to Be University, Vadodara, IND; 2 College of Physiotherapy, Sumandeep Vidyapeeth Deemed to Be University, Vadodara, IND

**Keywords:** cardiorespiratory fitness, fatigue, polycystic ovary syndrome, queens college step test, vo2 max

## Abstract

Introduction: Polycystic ovary syndrome (PCOS) commonly affects females of reproductive age in India, causing hormone imbalances, irregular menstrual cycles, high testosterone levels, and cysts. It is a chronic illness with no known cure, but medication, lifestyle modifications, and fertility treatments can help in managing some symptoms. In addition to the above irregularities, many physical symptoms, like fatigue and reduced functional capacity (maximal oxygen uptake, or VO_2_ max), commonly affect the day-to-day activities of these patients. The degree of impact of PCOS on functional capacity is still underexplored.

Objectives: The objective of the study was to assess the VO_2_ max and fatigue level of young females with PCOS.

Methodology: A cross-sectional observational study was conducted in which a total of 37 young females from health science institutions with PCOS aged 18-24 years participated in the study. The Fatigue Severity Scale (FSS) was used to evaluate the fatigue level of females with PCOS. The VO_2_ max of the participants was estimated using the Queens College step test (QCST). The Wilcoxon signed rank test and Spearman’s rank correlation coefficient were used to analyze the data.

Results: The mean age of the participants was 20.64+1.35 years. The participants' mean VO_2_ max was 35.25 mL/kg/min, which is lower than the typical average of 36-45 mL/kg/min. This implies that compared to healthy individuals, females with PCOS had decreased cardio-respiratory fitness (CRF) levels. There was no statistically significant correlation between the duration of PCOS, fatigue, and VO_2 _max. The mean fatigue score of 39.56 indicates that the majority of patients experienced above-average levels of fatigue (p > 0.05).

Conclusion: PCOS does not significantly impact VO_2_ max in young females, although they showed mildly lower VO_2_ max and mild to moderate fatigue levels compared to healthy individuals. Further research is needed to assess the impact of chronic PCOS on these variables.

## Introduction

Polycystic ovary syndrome (PCOS), also known as Stein-Leventhal syndrome, is a gynecological endocrinopathy that affects females of reproductive age [1,2]. It usually starts during adolescence, but symptoms may fluctuate over time. Global prevalence estimates range from 5% to 20% on the basis of the diagnostic criteria and the population studied. In Western nations such as the United States and Europe, the prevalence is usually between 6% and 10%. In Southeast Asia, such as China and Indonesia, prevalence rates have been reported similarly, ranging from 6% to 10%. In India, on the other hand, the prevalence of PCOS is slightly higher at 9% to 22%, with a significant increase in cases reported in urban regions [1]. This increase is largely attributed to lifestyle shifts, including unhealthy eating habits, sedentary lifestyles, and chronic stress, in combination with significant genetic and environmental factors [2]. Furthermore, the increasing incidence of PCOS in young Indian females underscores the importance of early detection and effective management.

PCOS is a heterogeneous and complex endocrine disorder that is phenotypically characterized by anovulation, hyperandrogenism, and polycystic ovaries [3]. The syndrome is frequently associated with several metabolic and endocrine abnormalities, such as hyperinsulinemia, disturbed glucose metabolism, and dyslipidemia, which overlap with metabolic syndrome features. As a result, patients with PCOS are at high risk for associated comorbidities such as obesity, type 2 diabetes mellitus, depression, and cardiovascular disorders (CVDs). Furthermore, an inflammatory condition, as reflected by increased levels of C-reactive protein (CRP), as well as increased oxidative stress, is also typically observed in such cases, further contributing to the pathophysiology of the condition [3].

Most PCOS-affected females also have an elevated body mass index (BMI) or clinical obesity, a disorder that exacerbates androgen secretion, disrupts metabolic function, and often results in infertility. A possible association between increased insulin production and PCOS has been confirmed. Other symptoms of PCOS are excess facial or body hair, hair loss on the scalp, acne, or high levels of testosterone in the blood [4]. In addition to its physical symptoms of infertility and obesity, PCOS also leads to anxiety, depression, and negative body image [4,5].

Certain lifestyle options can exacerbate the effects of PCOS and increase the risk of long-term complications. For example, poor dietary habits can exacerbate insulin resistance, a major issue in individuals with PCOS, especially if they are high in processed foods, white sugar, and unhealthy fats [6]. Chronic stress also increases cortisol levels, influencing disease progression [7]. PCOS females have increased risks of endometrial hyperplasia, infertility, and endometrial cancer, as well as pregnancy complications, including gestational diabetes and preeclampsia [6,8,9]. Obesity may exacerbate these issues because excess body fat exacerbates the hormonal imbalances characteristic of PCOS and worsens insulin resistance. Cardiovascular risk is substantially increased by related hypertension, dyslipidemia, and hyperinsulinemia [10]. The associated high androgen levels can cause skin and hair symptoms, whereas insulin resistance tends to result in acanthosis nigricans [11].

The confirmed relationship between PCOS and metabolic disorders, such as obesity, hypertension, dyslipidemia, and hyperinsulinemia, significantly enhances long-term risk for CVDs [11]. Screening for metabolic risk factors early and encouraging regular exercise are fundamental elements of overall management.

PCOS is also associated with a greater incidence of psychiatric disorders, such as depressive and anxiety symptoms. However, long-term emotional distress continues in adolescents after the fourth decade. Females with PCOS suffer from disordered eating and body image distress, which can interfere with weight loss. Screening routinely and interventions such as cognitive behavioral therapy are very important for controlling these disorders [12].

However, the effect of PCOS on the level of fatigue and maximal oxygen uptake (VO_2_ max) is still largely unknown. There are several lines of evidence suggesting that women with PCOS have a significantly higher rate of cardiovascular risk factors than females who otherwise remain healthy [6,13]. VO_2_ max is a widely used index of cardio-respiratory fitness (CRF) and is an independent, powerful predictor of functional capacity [13].

The fatigue is assessed by the Fatigue Severity Scale (FSS), developed in 1989, which is a nine-item scale that measures the severity of fatigue and its effect on a person's activities and lifestyle in patients with a variety of disorders. This scale was originally devised for people with multiple sclerosis (MS) or systemic lupus erythematosus. However, it can be used to assess the severity of fatigue in a variety of diagnoses, also validated for use in the PCOS population [14,15].

Impaired VO_2_ max in females with PCOS is an important concern because it is linked to many metabolic and cardiovascular abnormalities. Females with PCOS frequently exhibit decreased VO_2_ max, reflecting an impaired capacity of the cardiovascular and respiratory systems to effectively supply and utilize oxygen with exercise. This impairment is compounded by various factors, such as insulin resistance, obesity, and chronic inflammation [16]. Autonomic dysfunction also occurs, further impairing VO_2_ max. Elevated body fat, especially visceral fat, places a greater burden on the cardiovascular system, further worsening the impairment of VO_2_ max [17,18]. Decreased exercise tolerance and faster fatigue are frequently reported.

VO_2_ max is defined as the capacity of the lung, heart, and circulatory system to deliver oxygen to muscles during prolonged physical activities. Whereas VO_2_ max testing is the gold standard for precise measurement, its need for specialized equipment makes it less clinically useful [19,20]. Submaximal cycle ergometry tests, such as the Astrand-Rhyming and Young Men’s Christian Association (YMCA) protocols, provide approximations of VO_2_ max on the basis of heart rate response and are less expensive and more feasible for clinical use. Field tests such as the 1.5-mile run and the 12-minute Cooper test are inexpensive, easy alternatives for the estimation of VO_2_ max. The Queens College Step Test (QCST) is especially suitable for youths because it is low-impact, short, and easy to administer [21]. The QCST is a three-minute, submaximal cardiovascular endurance test using a 16.25-inch (41.3 cm) step, conducted at 22 steps/min for females and 24 steps/min for males. It estimates VO_2_ max by measuring heart rate recovery immediately after exercise. Because the risk of impaired VO_2_ max and consequent CVDs, and the fatigue burden are high, it is clinically important to have a better understanding of the cardio-respiratory consequences of PCOS [22,23].

Therefore, a comprehensive understanding of the consequences of PCOS on VO_2_ max is critical for improving patient outcomes. Hence, the primary purpose of this study was to evaluate fatigue with the FSS and VO_2_ max with QCST in young females diagnosed with PCOS.

## Materials and methods

This cross-sectional observational study was carried out over a 10-month period (from January 2024 to October 2024) at the College of Physiotherapy, Sumandeep Vidyapeeth Deemed to be University (SVDU), Vadodara, Gujarat, India, and was approved by the Sumandeep Vidyapeeth Institutional Ethical Committee (SVIEC) (SVIEC/ON/phys/BNMPT22/Oct/23/19). It was later registered with the Clinical Trial Registry India (CTRI) with the reference number CTRI/2024/01/061294. After the CTRI registration, from January 2024 onwards, young female students of the university, diagnosed with PCOS, were recruited through convenience sampling due to the exploratory nature of this study. The sample was not randomly selected; therefore, the findings may not be generalized to the broader population.

The sample size for this observational study was calculated using a margin of error of 15.4%, relying on a prevalence of 77% in the Indian population. The sample size was calculated using the following standard formula: $N = \frac{4pq}{L^2}$. The determined sample size was 30. Ultimately, 37 patients were recruited to increase the accuracy. The high margin of error was chosen to align with the exploratory nature of the study and the practical constraints of clinical recruitment for PCOS patients meeting the Rotterdam criteria.

The diagnosis of PCOS was made by the endocrinologist at a university multi-specialty hospital, based on the modified Rotterdam criteria, which requires at least two of the following: Oligo/anovulation, hyperandrogenism, or polycystic ovarian morphology, confirmed via clinical history, hormonal tests, and ultrasound results [1,15]. The young participants with stable vital signs were included, as this age group represents the early reproductive years, ensuring a stable presentation of PCOS-related symptoms for accurate assessment [15]. The participants were informed about the type and purpose of the study and were asked for their consent. After getting their consent, a participant information sheet was provided to each participant explaining the details of the study. Later, they were screened for eligibility as per the inclusion and exclusion criteria. Participants aged between 18 and 24 years, diagnosis of PCOS by endocrinologist with modified Rotterdam criteria were recruited, whereas participants with any neurological/cardio-respiratory/musculoskeletal problems that hinder the performance of the test and participants with other androgen excess disorders, such as nonclassical congenital adrenal hyperplasia and pregnancy at the time of recruitment, engaged in structured physical activity for the last six months were excluded from the study [15]. The information regarding medication use, usage of oral contraceptive pills, physical activity levels, smoking status, and alcohol use of the participants was collected at the time of recruitment.

Firstly, fatigue severity was evaluated using the FSS, a self-reported questionnaire given to all participants prior to the QCST performance, as it measures the fatigue level linked to PCOS. The FSS questionnaire contains nine questions that rate the severity of fatigue symptoms. Participants were asked to read each question and circle a number from 1 to 7, based on how accurately it reflects their condition during the past week and the extent to which they agree or disagree that the question applies to them [14]. The scale was clearly explained to all the participants in a simplified manner to minimize possible misinterpretation and ensure data accuracy. A score of ≥36 out of a total score of 63 indicates the presence of significant, severe fatigue. A score of <36 generally indicates normal levels of fatigue, or fatigue that does not significantly interfere with daily life.

Following the assessment of fatigue, on the same day, the submaximal QCST was performed to estimate VO_2_ max [24]. Prior to the test, participants were given adequate rest, and their baseline physiological variables, i.e., resting heart rate, respiratory rate, and blood pressure, were measured once in the supine position before the performance of the test. The QCST procedure was explained in detail and demonstrated. Participants performed the test on a 16.25-inch-high step, stepping up and down (one foot up, second foot up, first foot down, second foot down) in a four-count cadence, guided by a metronome, for a total duration of three minutes. The metronome was set to 88 beats per minute (bpm) for all participants (22 cycles/min) during the test. Immediately after the test, the participants were instructed to sit, and the post-test heart rate was measured by manually counting pulse at the radial artery between the 5th and 20th seconds, which was multiplied by four to convert to beats/min. The estimated VO_2_ max in mL/kg/min was then calculated using the following formula [24]:

$VO_{2\max} \; (\mathrm{ml/kg/min}) = 65.81 - (0.1847 \times \text{heart rate (bpm)})$

The estimated VO_2_ max was then categorized into different fitness categories, i.e., poor (<34.41), below average (35.15-34.41), average (36.63-35.15), above average (41.98-36.63), and excellent (>41.98) [25].

A Queen’s College step bench, stopwatch, metronome, sphygmomanometer, and pulse oximeter were the tools used for the performance of the test.

The statistical analysis was done using IBM SPSS Statistics for Windows, Version 28 (Released 2021; IBM Corp., Armonk, New York, United States)​​​​​​. The Wilcoxon signed rank test was used to assess the pre and immediate post-vitals of QCST. The relationship between the duration of PCOS (in years) and VO_2_ max categories was assessed using Spearman’s rank correlation coefficient. This non-parametric test was selected due to the ordinal nature of the aerobic capacity categories. A p-value of < 0.05 was considered statistically significant.

## Results

**Figure 1:**
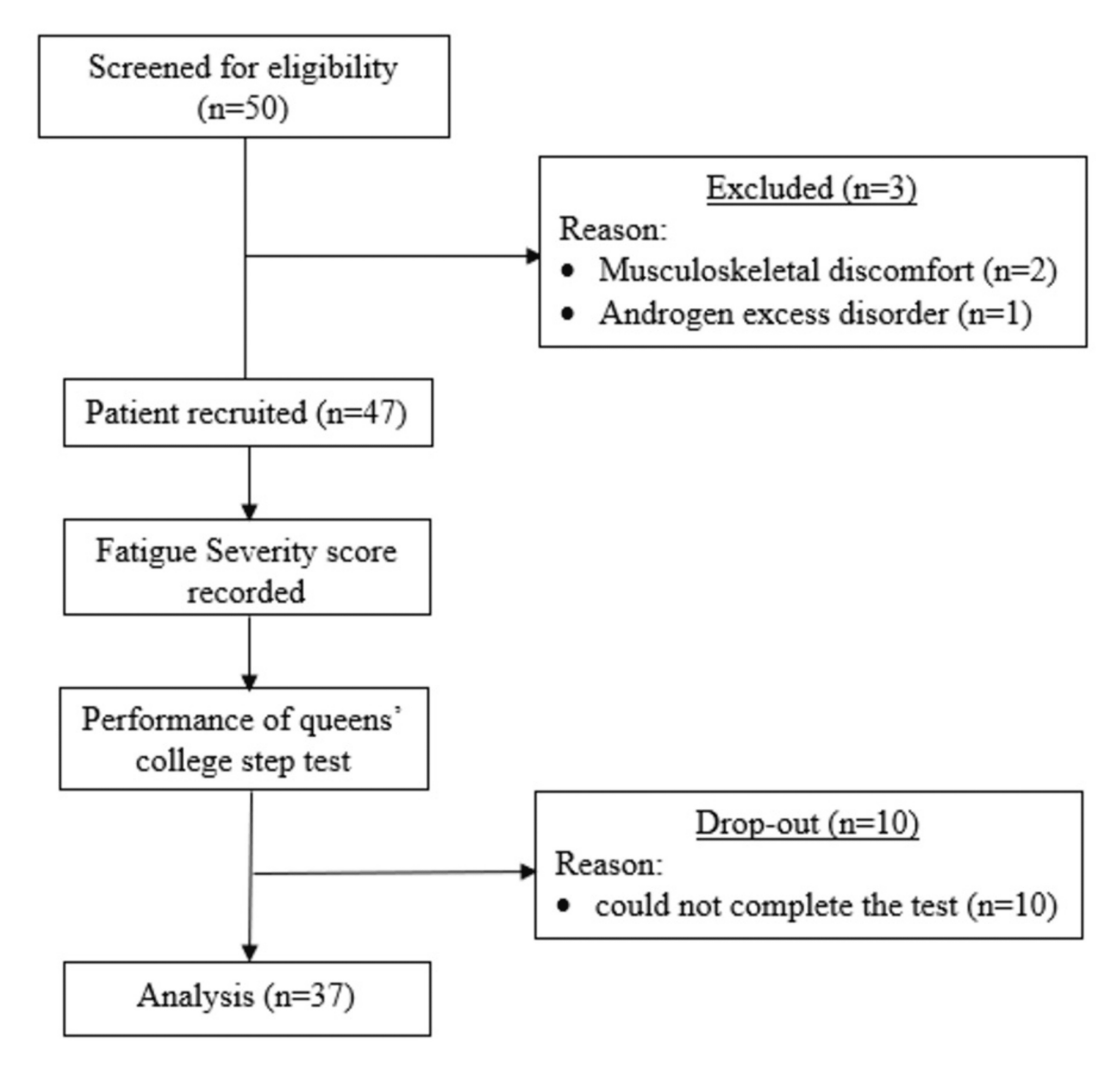
Flowchart showing recruitment of the study

Initially, a total of 50 participants from Sumandeep Vidyapeeth Deemed to Be University were screened for eligibility. During the initial screening, three participants were then excluded because of nonadherence to the inclusion criteria: two participants (n=2) were excluded because of musculoskeletal discomfort, and one participant (n=1) was excluded because of a preexisting androgen excess disorder. The remaining 47 participants were included in the study. Figure 1 shows the participant recruitment description. Subjective fatigue levels were then calculated via the FSS. The functional capacity, measured by the estimated VO_2_ max, was assessed via QCST. During the test, 10 participants were unable to complete the test and were excluded from the final analysis. Participants unable to finish the test are excluded for reasons of safety and ethical considerations. Ethically, cessation was necessary to prevent cardiovascular overexertion in participants having >15 respiratory distress in a Borg Scale rating. Additionally, the QCST is a submaximal protocol that uses recovery heart rate to estimate VO_2_ max, so if participants cannot finish the three-minute test, the physiological "steady state" isn't achieved, rendering the recovery heart rate an unreliable measure for the standard formula. Therefore, the data of 37 participants were included for the final analysis. Table 1 shows baseline characteristics of the participants.

**Table 1:** Baseline demographic data of the participants PCOS: polycystic ovary syndrome; FSS: Fatigue Severity Scale; VO_2_ max: maximal oxygen uptake

Variables (n=37)	Mean	Standard deviation
Age (years)	20.64	1.35
Height (meters)	1.58	0.06
Weight (kg)	60.89	9.38
BMI (kg/m^2^)	24.27	3.34
Diagnosed with PCOS since (months)	28.86	13.32
Heart rate (beats/min)	79.91	6.07
Blood pressure systolic (mmHg)	116.5	6.32
Blood pressure diastolic (mmHg)	77.19	6.1
Respiratory rate (breaths/min)	19.18	2.76
VO_2_ max (mL/kg/min)	35.25 (range 32.60 to 38.85)	2.60
Fatigue (FSS)	39.56 (range 31.02 to 41.32)	8.37

Differences in vital parameters before and after the QCST were analysed by the Wilcoxon signed-rank test (Table 2). The results demonstrated that the p-values for all vital signs were less than 0.05, indicating that the observed differences were statistically significant.

**Table 2:** Analysis of pre- and post-values (Queen’s College step test) using the Wilcoxon signed-rank test HR: heart rate; SBP: systolic blood pressure; DBP: diastolic blood pressure; RR: respiratory rate

Variables	Pre-vitals		Post-vitals		Z value	P-value
	Mean ± SD	Std. error of mean	Mean ± SD	Std. error of mean		
HR (beats/min)	79.91 ± 6.07	0.99	161.1 ± 29.13	4.78	-5.253	<0.00001
SBP (mmHg)	116.5 ± 6.32	1.03	127.29 ± 5.01	0.82	-5.318	<0.00001
DBP (mmHg)	77.19 ± 6.1	1.00	88.97 ± 5.09	0.83	-4.758	<0.00001
RR (breaths/min)	19.18 ± 2.76	0.45	27.08 ± 2.92	0.48	-5.373	<0.00001

The correlation between the duration of diagnosis of PCOS and VO_2_ max categories is presented in Table 3. Spearman’s rank correlation, which measures the strength of the relationship between PCOS duration and VO_2_ max, is calculated. There was no statistically significant correlation between the duration of PCOS and VO_2_ max (r=0.091, p=0.594). These findings suggest that the duration a patient has lived with PCOS does not directly influence their functional capacity in this study population.

**Table 3:** Correlations between duration of PCOS and functional capacity PCOS: polycystic ovary syndrome; VO_2_ max: maximal oxygen uptake

Diagnosed with PCOS since (months)	VO_2_ max categories				r value	P-value
	<34.41 (Poor)	34.41-35.15 (Below average)	35.16-36.63 (Average)	36.64-41.98 (Above average)		
<12 months	1	2	1	1	0.091	0.594
12-36 months	10	3	5	8		
36-48 months	1	2	1	0		
>48 months	0	0	1	1		

**Figure 2:**
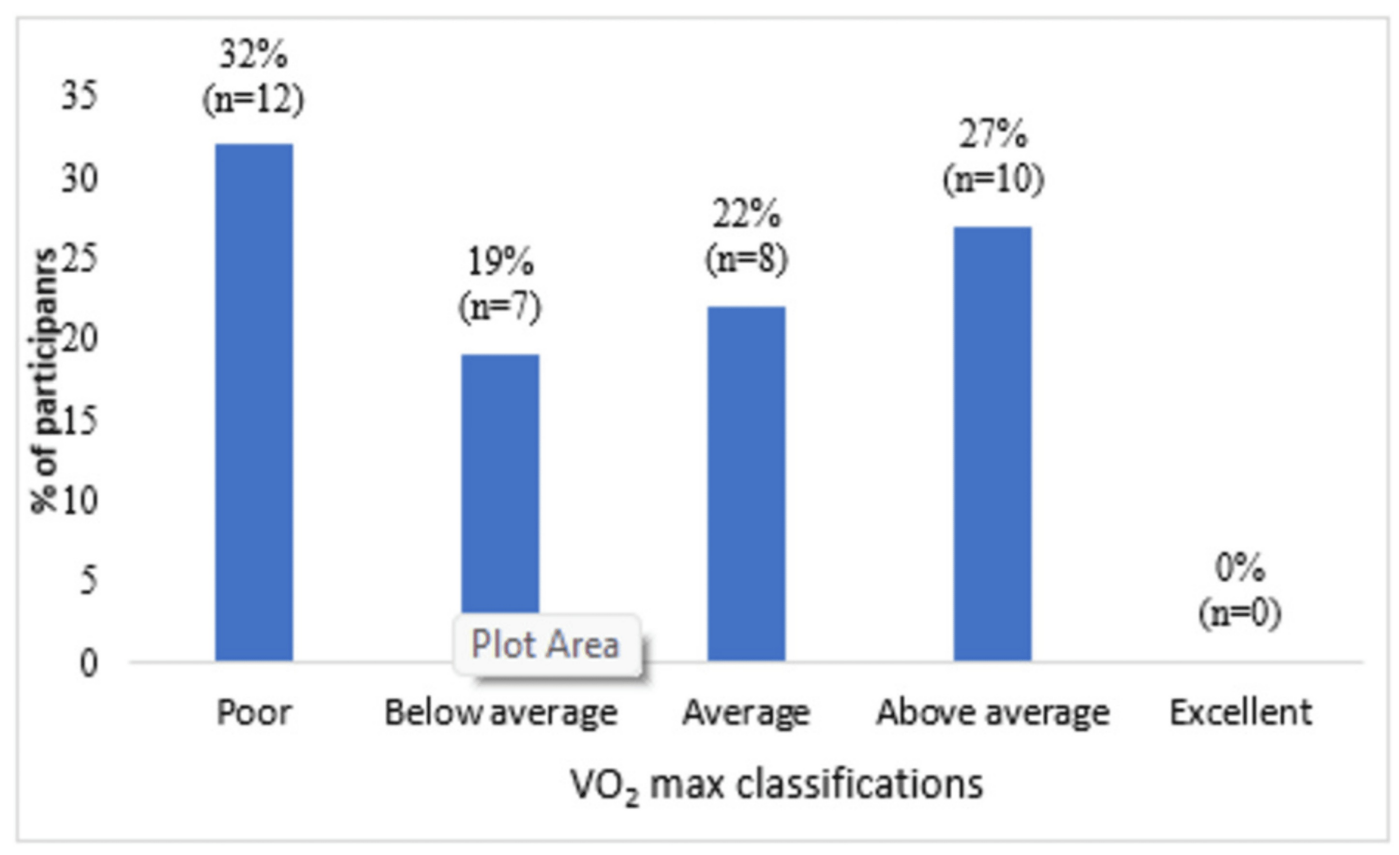
VO2 max of participants according to fitness category VO_2_ max: maximal oxygen uptake

Figure 2 illustrates the VO_2_ max distribution among the participants in the study. Five classifications are presented: poor, below average, average, above average, and excellent. A total of 12 participants (32%) exhibited poor VO_2_ max, seven participants (19%) were below average, eight participants (22%) had average, and 10 participants (27%) demonstrated above average VO_2_ max levels. None of the participants exhibited excellent VO_2_ max. The data shows that most participants exhibited average or below-average VO_2_ max values, with none achieving excellent scores.

The fatigue levels of the participants, as measured with the FSS is shown in Table 4. The mean fatigue level is 39.56, the median is 38, and the mode is 37. This suggests that most of the participants had a fatigue score above average (moderate) but with significant variability, as indicated by the standard deviation of 8.37.

**Table 4:** Mean, median, mode and standard deviation of the FSS FSS: Fatigue Severity Scale

Variable	Mean	Median	Mode	SD	SD error of the mean
FSS	39.56	38	37	8.37	1.37

## Discussion

The present study examined the major indicators of health and levels of functional capacity, namely, VO_2_ max, in a group of young females with PCOS, which is a prevalent endocrine disorder of reproductive-aged women that causes a variety of manifestations, such as irregularities in menstruation, infertility, hyperandrogenism, and profound metabolic abnormalities [26].

The mean BMI of the participants in this study was 24.27 kg/m^2^, which places the average subject in the overweight category on the basis of the Asia-Pacific BMI classification. This is extremely relevant, since both weight gain and difficulty with healthy weight maintenance are hallmark problems in individuals with PCOS and are commonly driven by insulin resistance, which strongly affects cardiovascular risk factors [27]. The high BMI observed in this sample indicates that many of the participants are already experiencing metabolic dysfunction, which adds to the cardiovascular load, as evidenced by possibly elevated heart rate and blood pressure. These results highlight the importance of frequent monitoring of cardiovascular health in young women with PCOS for early risk detection [27].

The mean VO_2_ max of the participants was 35.25 mL/kg/min. The normative VO_2_ max values for healthy young women are usually approximately 36-45 mL/kg/min, which indicates that the CRF level in women with PCOS is less than that in healthy individuals [28]. This finding is in keeping with current evidence, invariably showing that women with PCOS tend to have lower VO_2_ max, which is presumably the result of multifactorial impairments, such as insulin resistance, obesity, and lower habitual physical activity [28]. This finding is also supported by other research that revealed that young, obese women with PCOS had lower maximal oxygen intake and peak exercise load than healthy controls did, which is commonly attributed to this decline in insulin resistance and mitochondrial malfunction [29]. However, according to a meta-analysis by Cirone et al. (2024), although VO_2_ max is decreased in PCOS patients, its impact on muscle strength and endurance is less certain [30].

The mean FSS score was 39.56, suggesting that, on average, the participants experienced moderate to severe levels of fatigue. This is reflected by the median score of 38 and the mode score of 37, showing that the majority of participants reported similar levels of fatigue severity, with few individuals reporting extreme levels of fatigue. This variability suggests that while many participants with PCOS experience fatigue at moderate to high levels, some participants may experience either milder or more severe fatigue.

Moreover, the variability in FSS scores suggests that the impact of fatigue in patients with PCOS may be influenced by other factors, such as age, lifestyle, associated comorbidities, and mental health status. Women with PCOS frequently report fatigue, which is often associated with hormonal imbalances. These factors can lead to decreased energy levels and negatively impact overall well-being. However, the range of FSS scores in this study also indicates that not every female with PCOS experiences fatigue to the same intensity, which emphasizes the importance of personalized approaches in managing fatigue for individuals with this condition [6,13,14].

In total, the results highlight that young females with PCOS are more likely to be overweight and have suboptimal VO_2_ max than the healthy population, which places them at a premature and persistent risk for CVD. The absence of a significant correlation between VO_2_ max and PCOS duration implies that complications with early onset are driven by more associated metabolic factors (i.e., BMI) than by the time between diagnosis. The high prevalence of fatigue not directly related to VO_2_ max emphasizes the need to address the psychological and metabolic nature of fatigue separately from fitness training. Longitudinal studies should be the focus of future research to conclusively determine the course of CRF loss and the long-term effectiveness of specific lifestyle interventions as countermeasures for cardiovascular risk among this at-risk population.

Although useful preliminary findings were generated, the research has several limitations. First, the demographic composition of the cohort means that the findings are limited in terms of generalizability because the predictive value of VO_2_ max for long-term CRF can be lower in this age group than in older groups.

The exclusion of participants unable to complete the QCST may introduce attrition bias, as these individuals likely represent a subgroup with lower baseline functional capacity. Excluding them may have resulted in an overestimation of the baseline fitness levels and treatment effects within the cohort. Future studies should consider a tiered testing approach, utilizing the 6-Minute Walk Test (6MWT) for participants unable to perform the QCST to ensure a more inclusive representation of the patient population.

Another limitation is that the participant recruitment was done using convenience sampling. To address these limitations, future research should aim to employ stratified random sampling across multi-center sites, including both rural and urban healthcare facilities, to validate these findings across a more diverse spectrum of PCOS phenotypes.

While these findings provide valuable preliminary data on VO_2_ max on PCOS population, they may not be fully representative of the broader population because of choosing the convenience sampling, and larger multi-center studies are required to validate these trends with greater precision.

## Conclusions

This preliminary research emphasizes the cardiovascular challenges faced by young females with PCOS. This exploratory cross-sectional study suggests that young women with PCOS demonstrate moderate fatigue levels and mildly reduced CRF. No significant association was observed between the duration of PCOS and VO_2_ max. Larger controlled studies are required to determine whether PCOS independently impairs CRF.
